# Supplementary material for: Growth Trajectories during the First 6 Years in Survivors Born at Less Than 25 Weeks of Gestation Compared with Those between 25 and 29 Weeks
Source: J Clin Med. 2022 Mar 4;11(5):1418. doi: 10.3390/jcm11051418 (PMC8911231; doi:10.3390/jcm11051418)
Supplement: Supplementary file 1 [file jcm-11-01418-s001.zip › jcm-1616463-supplementary.pdf]

Journal of
Clinical Medicine

MDPI

Table S1. Anthropometric indices at birth and 6 years of the children with and without follow-up.

		With Follow-Up		Without Follow-Up		*p* (Male)	*p* (Female)
		Male	Female	Male	Female		
22–24 weeks	n	16	28	13	13		
	Gestational age (weeks)	24.2 ± 0.6	24.0 ± 0.6	24.1 ± 0.8	23.8 ± 0.7	0.99	0.55
	Weight (g)	635.4 ± 127.3	605.2 ± 93.0	659.5 ± 103.7	570.4 ± 102.3	0.50	0.18
	Weight z-scores	−0.4 ± 1.2	−0.0 ± 0.8	0.0 ± 1.2	−0.2 ± 1.2	0.35	0.47
	Length (cm)	29.7 ± 2.2	29.7 ± 1.7	30.4 ± 1.8	29.2 ± 1.8	0.32	0.31
	Length z-scores	−0.3 ± 0.9	−0.2 ± 0.7	0.1 ± 0.9	−0.3 ± 0.9	0.18	0.65
	HC (cm)	21.6 ± 1.0	21.0 ± 1.1	21.6 ± 1.5	21.0 ± 1.1	0.99	0.92
	HC z-scores	0.1 ± 0.6	−0.2 ± 0.7	0.1 ± 1.0	−0.2 ± 0.8	0.73	0.76
	Small for gestational age	1 (6.3)	1 (3.6)	1 (7.7)	1 (7.7)	0.92	0.61
25–29 weeks	n	84	59	101	88		
	Gestational age (weeks)	27.6 ± 1.5	27.7 ± 1.5	27.7 ± 1.5	27.9 ± 1.5	0.53	0.47
	Weight (g)	936.6 ± 250.7	878.3 ± 268.4	1006.4 ± 228.3	990.4 ± 235.5	0.06	0.01
	Weight z-scores	−1.0 ± 1.2	−1.2 ± 1.3	−0.6 ± 1.2	−0.5 ± 1.1	0.01	<0.01
	Length (cm)	34.6 ± 3.0	34.0 ± 3.7	35.3± 2.7	35.3 ± 3.0	0.16	0.05
	Length z-scores	−0.4 ± 1.1	−0.8 ± 1.2	−0.2 ± 1.2	−0.3 ± 1.1	0.16	0.01
	HC (cm)	24.9 ± 2.2	24.1 ± 2.3	25.1 ± 1.9	25.0 ± 1.9	0.30	0.03
	HC z-scores	−0.0 ± 0.9	−0.5 ± 0.9	0.1 ± 0.9	−0.2 ± 0.8	0.36	<0.01
	Small for gestational age	17 (20.2)	18 (30.5)	15 (14.9)	9 (10.2)	0.33	<0.01

Data are presented as the number (percentage) of subjects. * $p < 0.05$, ** $p < 0.01$, compared with the 22–24 weeks group.
